# Large Deletions, Cleavage of the Telomeric Repeat Sequence, and Reverse Transcriptase-Mediated DNA Damage Response Associated with Long Interspersed Element-1 ORF2p Enzymatic Activities

**DOI:** 10.3390/genes15020143

**Published:** 2024-01-23

**Authors:** Kristine J. Kines, Mark Sokolowski, Cecily DeFreece, Afzaal Shareef, Dawn L. deHaro, Victoria P. Belancio

**Affiliations:** 1 Department of Structural and Cellular Biology, Tulane School of Medicine, Tulane Cancer Center, New Orleans, LA 70112, USA; 2 Department of Biology, Xavier University of Louisiana, New Orleans, LA 70125, USA

**Keywords:** LINE-1, ORF2p, retroelements, DNA damage, H2AX, telomeres, genomic instability

## Abstract

L1 elements can cause DNA damage and genomic variation via retrotransposition and the generation of endonuclease-dependent DNA breaks. These processes require L1 ORF2p protein that contains an endonuclease domain, which cuts genomic DNA, and a reverse transcriptase domain, which synthesizes cDNA. The complete impact of L1 enzymatic activities on genome stability and cellular function remains understudied, and the spectrum of L1-induced mutations, other than L1 insertions, is mostly unknown. Using an inducible system, we demonstrate that an ORF2p containing functional reverse transcriptase is sufficient to elicit DNA damage response even in the absence of the functional endonuclease. Using a TK/Neo reporter system that captures misrepaired DNA breaks, we demonstrate that L1 expression results in large genomic deletions that lack any signatures of L1 involvement. Using an in vitro cleavage assay, we demonstrate that L1 endonuclease efficiently cuts telomeric repeat sequences. These findings support that L1 could be an unrecognized source of disease-promoting genomic deletions, telomere dysfunction, and an underappreciated source of chronic RT-mediated DNA damage response in mammalian cells. Our findings expand the spectrum of biological processes that can be triggered by functional and nonfunctional L1s, which have impactful evolutionary- and health-relevant consequences.

## 1. Introduction

Long Interspersed Element-1 (L1) retrotransposons are currently active in most mammalian genomes [1,2,3,4,5,6,7]. L1 expression is reported to occur in the germ line, during embryogenesis, and in most adult somatic tissues [8,9,10,11,12]. The significant upregulation of L1 mRNA and protein expression, as well as retrotransposition, has been observed in human tumors and induced pluripotent stem cells (iPSCs) [7,13,14,15,16,17,18,19,20]. This increase is attributed to the reactivation of the L1 promoter associated with genome-wide hypomethylation and epigenetic reprogramming [21,22,23,24]. The epigenetic control of L1 promoter activity is likely one of the reasons underlying cell type-, organ-, sex-, and age-specific differences in the spectrum of endogenous L1 loci expressed in human, mouse, and rat organs [9,25,26,27]. This is further supported by a comparative analysis of expressed and silent L1 loci in human cell lines, which identified specific epigenetic marks associated with expressed L1 loci and the requirement of long-range interactions with enhancers for optimal L1 promoter function [28,29]. These analyses have also established that both retrotransposition-competent and -incompetent endogenous L1 loci are expressed [9,25,26,27]. Studies of retrotransposition-competent L1 loci have dominated the field from the time of its conception, with only a handful of studies recognizing the potential of expressed retrotransposition-incompetent L1 loci to impact host genome stability or cell function [30,31,32,33,34].

**Figure 1:**
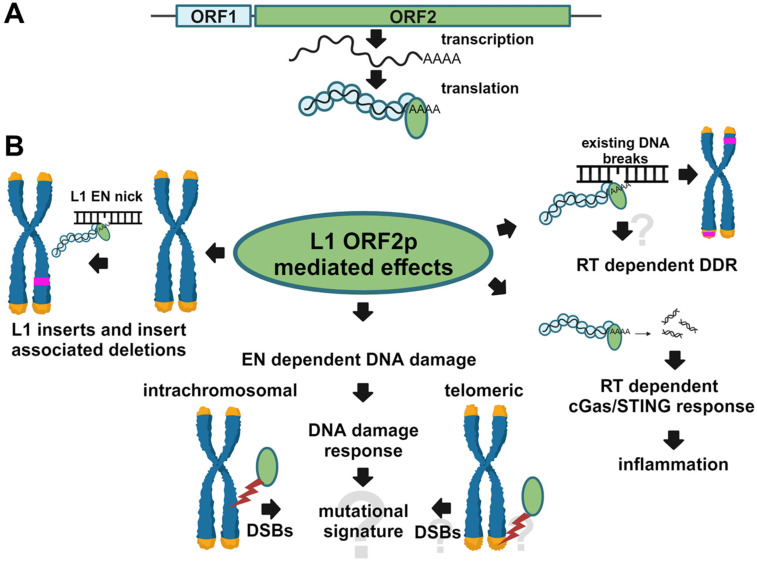
An overview of the L1 replication cycle and consequences of L1 ORF2p enzymatic functions. (**A**) Pol-II-driven transcription of a full-length functional L1 locus produces a full-length bicistronic mRNA containing open reading frames (ORFs) encoding ORF1 (blue) and ORF2 (green) proteins. The two proteins and the L1 mRNA assemble into L1 ribonucleoprotein (RNP) complexes capable of mobilization. The ORF2p contains an endonuclease (EN) domain that initiates L1 integration by nicking genomic DNA. It also contains a reverse transcriptase (RT) domain, which is responsible for DNA primer-mediated cDNA synthesis during L1 integration. (**B**) Expression of functional L1 in human cells results in de novo L1 inserts (pink), which sometimes cause large genomic rearrangements (left). This process relies on both EN and RT functions. The L1 expression also leads to the formation of EN-dependent DNA double-strand breaks (shown with a red bolt), which trigger DNA damage response (center). Other than L1 inserts, the spectrum of mutations resulting from these DSBs and whether L1 EN can cut telomeric sequences is not known. Among RT-dependent outcomes are EN-independent L1 intrachromosomal and telomeric integrations (shown with pink bands, bottom right) and inflammatory response triggered by cGas/STING activation by RT-generated cDNA. Whether L1 RT-mediated cDNA synthesis can trigger DNA damage response (DDR) is not known (top right). Question marks indicate unknown outcomes.

A full-length L1 locus, harboring an intact promoter in its 5’UTR, generates L1 mRNAs containing two open reading frames 1 and 2 (ORF1 and ORF2) (Figure 1A) (reviewed in [35,36]). L1 mRNA translation results in the production of ORF1 and ORF2 proteins (ORF1p and ORF2p), which are required for L1 retrotransposition [37,38,39,40,41,42,43,44,45,46,47,48]. ORF1p is a structural protein that performs obligatory functions involving trimerization, RNA binding, nucleic acid chaperon function [38,48,49,50], and contains amino acids involved in its nuclear localization [51]. ORF2p is a multifunctional protein containing endonuclease (EN) and reverse transcriptase (RT) enzymatic activities [42,43,52,53,54,55,56]. ORF2p also contains Cryptic, Z, and C-terminal regions, which do not possess any known enzymatic activities but are required for ORF2p function in retrotransposition [39,40,53,57,58]. The EN domain is structurally and functionally related to the cellular APE1 protein, which is an endonuclease that is essential for DNA repair via the BER pathway [43]. Based on the in vitro studies of the prototypic R2 element and L1 ORF2p, the EN domain of the ORF2p is responsible for the generation of DNA nicks used by the RT domain to initiate cDNA synthesis leading to the production of de novo L1 inserts [53,59,60,61,62]. ORF2p-dependent cDNA synthesis requires reverse transcriptase, Cryptic, Z, and C-terminal regions [40,42,47,53]. The process of cDNA synthesis by ORF2p has been linked to the induction of inflammatory response through generation of cytoplasmic L1 cDNA [63].

L1 contributes to genomic instability via retrotransposition and the generation of DNA double-strand breaks (DSBs) [64,65]. The outcome of L1 retrotransposition is de novo L1 inserts, some of which are mutagenic because they disrupt gene function or cause large deletions at their integration site [7,36,64,66,67] (Figure 1B). While efficient L1 retrotransposition relies on the function of both the endonuclease and the reverse transcriptase domains, experimental evidence shows that the two domains can function independently of each other and even outside of the context of the full-length ORF2p molecule [30,31,40]. When purified from bacteria, the EN domain cleaves DNA substrates in vitro and, when expressed in mammalian cells, causes significant DNA damage [32,43,68] (Figure 1B). The endonuclease-independent retrotransposition of defective L1 elements in mammalian telomeres [30] and in some DNA repair-deficient cells [31] has been reported (Figure 1B), demonstrating that the RT domain can function in the context of the ORF2p containing nonfunctional EN domain. Additionally, ORF2p deficient in EN function or completely lacking the EN domain supports residual levels of Alu retrotransposition in cell culture-based assays [40].

The involvement of the EN domain in causing DNA breaks and eliciting cellular DNA damage response (DDR) has been reported using the detection of phosphorylated H2AX, COMET, or toxicity assays [32,65,69,70]. The histone H2AX is a fairly common component of the chromatin of mammalian cells, with about 1 in 40 to 1 in 4 histones being H2AX, depending on the cell type [71]. Following a double-stranded break (DSB) in the DNA, H2AX phosphorylation occurs rapidly and spreads around the break site [71,72,73,74]. H2AX phosphorylation is used in clinical (reviewed in [75]) and in vitro (reviewed in [76]) studies to characterize the presence of DSBs. It is well established that L1 ORF2p and the endonuclease domain can cause DNA breaks and DNA damage response (Figure 1B) [32,65]. Mammalian cells have multiple DNA repair pathways that handle the repair of DSBs, leading to faithful or mutagenic repair, which produces small or large deletions/insertions or complex chromosomal rearrangements [77,78]. Whether any of these types of DSB misrepair happen at the L1-induced DSBs is not known (Figure 1B).

In addition to supporting residual retrotransposition without functional EN, the L1 RT domain has been linked to triggering an inflammatory response through generation of cytoplasmic cDNA (Figure 1B) [63]. The mechanism of this process is not completely understood in light of existing biochemical studies [62,79]. The proposed mechanism involves the promiscuous ability of the ORF2p RT to utilize various forms of nucleic acids as a primer for cDNA synthesis [53,63]. With many nonfunctional L1 loci retaining either functional EN or RT enzymatic activities being expressed in normal and cancer cells [9,26,27], gaining a better understanding of the potential of these loci to elicit a DNA damage response and their contribution to genomic instability is necessary.

The DNA damage caused by individual endogenously expressed L1 loci is difficult to reliably measure in vivo due to the lack of sensitive and quantitative approaches and very low expression levels of endogenous L1 elements [25,26]. Therefore, L1-associated DDR has been investigated using various versions of transiently transfected L1 or ORF2p expression plasmids [32,65,70]. A major disadvantage of this approach is that transient transfections themselves may cause a DDR-like response in mammalian cells [80,81], creating a background signal that often masks cellular response to the L1-associated damage. We capitalized on the reported inducible L1 system that eliminates the need for transient transfections to trigger measurable L1 expression [57]. Using this approach, we generated plasmids that express defective L1 elements, ORF2, and EN-containing truncated proteins harboring inactivating mutations within their EN and/or RT domains. Our findings demonstrate that the induced expression of either an EN-mutant full-length L1 element or an EN-mutant ORF2 leads to the accumulation of phosphorylated H2AX (γH2AX) signal. Our findings provide evidence that retrotransposition-incompetent L1 loci, specifically those that retain RT function, can contribute to the DDR, which may contribute to the observed RT-dependent inflammatory response triggered by L1. We also report a Thymidine Kinase Neomycin resistance fusion reporter cassette designed to capture novel types of L1-associated mutations. This system shows that exposure to functional L1 can lead to de novo genomic deletions that lack any hallmarks of L1 contribution. Finally, we report that the L1 endonuclease domain purified from bacterial cells can efficiently cut mammalian telomeric sequences in vitro, establishing the foundation for future studies involving its potential contribution to telomere shortening occurring during normal aging and short telomeres typically present in cancer cells.

## 2. Materials and Methods

### 2.1. Generation of L1-, ORF2-, and EN-Containing Plasmids

EN (D205A) (5′-CCTACAGCAAGATCGCCCACATCGTGGGCAG-3′ and 5′-CTGCCCACG ATGTGGGCGATCTTGCTGTAGG-3′)- and RT (D702A) (5′-GAGCCTGTTCGCCGCCGAC ATGATCGTGT-3′ and 5′-ACACGATCATGTCGGCGGCGAACAGGCTC-3′)-inactivating mutations were introduced into the previously reported L1 expression plasmid [57] using site-directed mutagenesis as previously described [32]. The human codon-optimized ORF2 sequence [57,70] was PCR-amplified using these L1-containing plasmids for subcloning to generate plasmids containing wild-type and mutant ORF2 sequences that can be used in the reported inducible system.

### 2.2. Cell Culture

FLP-In™-293 (Invitrogen, Carlsbad, CA, USA) cells were cultured in HyClone Dulbecco’s modified Eagle’s medium with 10% fetal bovine serum (Invitrogen) and maintained under 6% CO_2_ at 37 °C. Tet-on HEK 293 cells were cultured as previously described [32,57]. The cells were seeded (1 million cells per T25 flask) 16–18 h prior to induction with doxycycline (dox) or transfection (normal growth media was replaced 3 h post-transfection for all experiments) [57].

### 2.3. Generation of Doxycycline-Inducible 293T Cell Lines

Cell lines harboring wild-type or mutant L1-, ORF2-, and EN-containing plasmids were generated as previously described [57]. At least two independently generated inducible cell lines were used for each experiment. The induction of L1, ORF2, or ORF2-truncated fragments was initiated by doxycycline (1 µg/mL) treatment as described [57].

### 2.4. Protein Harvest for Western Blot Analysis

Cells were washed with 1X PBS (137 mM NaCl (Sigma S9888), 2.7 nM KCl (Sigma P4505), 10 mM Na2HPO4 (Sigma S3264) and 2 mM KH2PO4 (Sigma P9791), pH = 7.4) and harvested using 300 µL of TLB SDS (50 mM Tris, 150 mM NaCl, 10 mM EDTA, 0.5% sodium dodecyl sulfate, TritonX-100 0.5% *v*/*v*, Halt Protease inhibitor 10 µL/mL, phosphatase inhibitors 2 and 3 (Sigma, Livonia, MI, USA), pH = 7.2) per T25 flask. The total lysate samples were sonicated three times for 10 s at 12 watts RMS using a 3 mm wide Qsonica Microson homogenizer with Microson ultrasonic cell disruptor XL2000 (Microson, Brussels, Belgium). The samples were centrifuged at 21,130× *g* for 15 min at 4 degrees Celsius. The resulting supernatant was transferred to a new microcentrifuge tube. The protein concentration for each sample was determined using 595 nm wavelength OD values against a Bovine Serum Albumin (BSA) standard. An equal amount of protein (15–30 micrograms) was loaded for each sample.

### 2.5. Western Blot Analysis

For each sample, 15–20 µg of protein was combined with 2x Laemmli buffer to obtain the final concentration of 1X, 1.6 µL β-mercaptoethanol, and heated at 100 degrees Celsius for 5 min prior to loading. The samples were fractionated on a Bis-Tris 4–12% Midi gel or Novex Tris-Glycine 4% Mini gel (Invitrogen) and transferred to a nitrocellulose membrane (iBlot2 system: Invitrogen). The membrane was first incubated for 1 h in the 5% milk/PBS-Tween buffer (0.1% *v*/*v* Tween 20 (Sigma P2287), 137 mM NaCl (Sigma S9888), 2.7 nM KCl (Sigma P4505), 10 mM Na2HPO4 (Sigma S3264) and 2 mM KH2PO4 (Sigma P9791), pH = 7.4), and then overnight at 4 degrees Celsius with 1:5000 dilution of hORF1 (custom polyclonal rabbit antibody: TGNSKTQSASPPPK antibody [46] or 1:10,000 Flag-Tag-Specific (Sigma Monoclonal Mouse Anti-Flag M2: F3165) antibody, 1:100,000 γH2AX (Santa Cruz, CA, USA; sc-101696) antibody, or ORF2-specific antibodies [32] in 3% milk/PBS-Tween buffer. Following the overnight incubation, the membranes were washed for 5 min 3 times using PBS-Tween buffer and incubated at room temperature for 1 h with 1:5000 dilution of horseradish peroxidase-conjugated secondary antibodies (HRP-donkey anti-rabbit (Santa Cruz: sc2317)) in 3% milk/PBS-Tween buffer. The membranes were washed one time for 15 min with PBS-Tween buffer and then twice for 5 min using PBS-Tween buffer. The development was carried out using a 5 min incubation with a Bio-Rad Clarity Kit (Bio-Rad, Hercules, CA, USA). The signal was detected using a Chemi-Doc XRS+ Molecular Imager (Bio-Rad). anti-GAPDH antibodies (Santa Cruz: sc25778, 1:5000 dilution) were used to detect GAPDH using the incubation conditions described above.

### 2.6. LEAP Assay

Analysis of the RT function was performed using LEAP as previously described [40,79]. In short, HEK 293 cells were transiently transfected with indicated ORF2 expression plasmids; 48 h later cytoplasmic RNP-containing fraction was extracted and purified using sucrose gradient. The resulting RNP-containing pellet was used for testing the ORF2 RT-mediated cDNA synthesis using a primer specific to the 3′ end of the ORF2 expression plasmid and a primer specific to the LEAP primer.

### 2.7. Retrotransposition Assay

The retrotransposition assay was performed in HeLa cells as previously described using AluNeo reporter plasmid [82].

### 2.8. Analysis of L1-Induced Mutations

The TKNeo reporter cassette was synthesized by GenScript. An annotated TK/Neo reporter sequence is shown in Supplemental Materials (File S1). The resulting DNA was then cloned in the pEF5/FRT/V5-DEST destination vector of the Flp-In system (Invitrogen). An amount of 0.6 µg of this plasmid was co-transfected with 5.4 µg of the pOG44 Flp-recombinase plasmid expressing the Flp recombinase using 12 µL of Plus and 36 µL of Lipofectamine into the FRT containing HEK 293-FRT cells seeded 18 h prior (5,000,000 cells). Then, 48 h later, cells were reseeded, and hygromycin selection was initiated 24 h later. Homologous recombination between the FRT sites results in the site and direction-specific integration of a single copy of our TKNeo cassette into the cellular genome. Stable integration of our pEF5/FRT/V5-DEST TK/Neo cassette confers hygromycin resistance to the cells. Hygromycin-resistant HEK 293-FRT colonies were picked and expanded. Selection was used to select cells containing a single copy of the TK/Neo cassette. Their genomic DNA was used to screen clones containing TK/Neo cassette by site-specific PCR with primers complementary to the SV40 promoter and Hygromycin. Selected positive clones were expanded in the presence of neomycin to eliminate any cells that may have undergone any L1-induced or random mutagenesis during normal cell proliferation. 500,000–1,000,000 cells was seeded and transfected the next day with 0.4–1 µg of either ISce-I-, L1-, or ORF2p-expressing plasmids containing EN- or RT-inactivating mutations. An amount of 4–8 µL of Plus Reagent and 12 µL of Lipofectamine were used. Gancyclovir selection (25 µM) was initiated 2–4 days post-transfection and was maintained for 14 days or until cell collection.

### 2.9. Site-Specific PCR Analysis

500,000 Neomycin-resistant TKNeo cells were seeded in T75 flasks and transiently transfected ~18–20 h later with 0.5–2 µg of L1 or ISceI expression plasmids (4 µL of plus and 12 µL of Lipofectamine) to induce DSBs. Selection with 1 µM ganciclovir was initiated two to four days post-transfection and maintained for 2 weeks to allow for colony formation. DNA from L1 and ISceI exposed cells was collected at 2, 3, 4, 5, or 14 days after transfection. Site-specific PCR analysis was performed using 100 ng of DNA template, 5% DMSO, 1 µL of a forward and 1 µL of a reverse primer, and 45 µL of Platinum PCR Supermix. PCR cycling conditions: 94C for 1 min, [94C for 30 s, 55C for 30 s, 68C for 45 s] X 33 or 35 cycles, 72C for 10 min. The primers used for PCR are as follows:

pEF1355F primer (5′-GAGGGGTTTTATGCGATGGAGTTT-3′);

TK/Neo291R primer (5′-AGCAGTTGCGTGGTGGTGGTTTTC-3′);

pEF5F primer (5′-CGCCCACAGTCCCCGAGAA-3′);

200F primer (5′-CCAGTGTGGTGGAATTCTGCAGA-3′);

200R primer (5′-GCAGACGCATGTTGATGGCA-3′);

F3 primer (5′-CAGGGTTATTGTCTCATGAGCGGA-3′);

R3 primer (5′-ATGCGGCATCAGAGCAGATTGT-3′);

F6 primer (5′-GCTATGACTGGGCACAACAGACA-3′);

NeoR primer (5′-TGCCTCGTCCTGCAGTTCAT-3′).

Primer sequences are annotated in the TK/Neo reporter sequence in the Supplemental Materials, except for the F3/R3 primer pair because they are complementary to the sequence in the Flp-In cassette between the Amp gene and pEF5 promoter.

### 2.10. Endonuclease In Vitro Cleavage Assay

Endonuclease in vitro cleavage assay was performed as previously described using Escherichia coli (*E. coli*) purified L1 endonuclease domain [68]. Primer sequences L1 WT 5′-AGGCTTAAAATT AAAATTAAAATTAAAAAGGC-3′, TEL WT 5′-AGGCTTAGGGTTAGGGTTAGGGTTA GGGAGGC-3′, TEL MUT 5′-AGGCTCCGGGTCCGGGTCCGGGTCCGGGAGGC-3′ and their reverse complement sequences were used to generate double-stranded DNA substrate. Either the top or bottom strand was 5′ labeled with AlexaFluor 488 (IDT).

### 2.11. Assessment of Disease-Causing Mutations and the Number of L1 EN Sites in Human Genes

Gene sequences were extracted from the human genome assembly. The number of L1 endonuclease recognition sites was determined using the DNASTAR Lasergene program. The number of L1 sites per kb was determined using the following formula: total # of L1 sites/total length of a gene. The number of total disease-causing mutations for each gene was determined using The Human Gene Mutation database (HGMD) https://www.hgmd.cf.ac.uk/ac/index.php. (accessed on 117 October 2017)The percent of genomic deletions per gene was calculated using the following formula: (# deletions/# total mutations) X100. Pearson correlation was performed using GraphPad Prism.

## 3. Results

### 3.1. RT-Dependent Accumulation of γH2AX Signal upon Induction of L1 Expression

**Figure 2:**
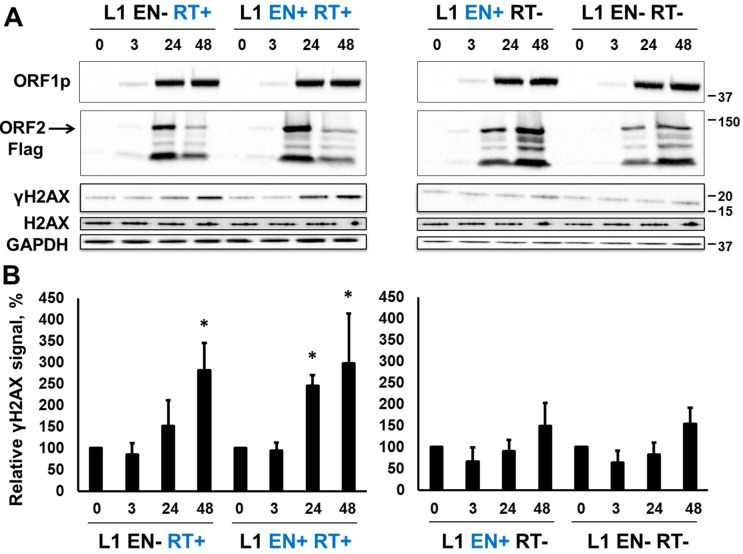
Expression of retrotransposition-incompetent L1 elements triggers DNA damage response. (**A**) Western blot analysis of L1 ORF1 and ORF2 protein expression and γH2AX and H2AX signal in HEK 293 cells at 3, 24, and 48 h post-induction of L1 expression with Doxycycline. EN+ (blue) and EN- correspond to either functional or mutant endonuclease domains. RT+ (blue) and RT- correspond to the functional and mutant reverse transcriptase domain. Anti-ORF1-specific Ab was used to detect ORF1p. Anti-Flag-tag-specific antibodies were used to detect ORF2 protein. A band corresponding to the full-length ORF2p is indicated by the arrow. Anti-phosphoH2AX- and H2AX-specific antibodies were used to detect the γH2AX and H2AX signals, respectively. (**B**) Quantitation of the γH2AX signal relative to the 0-time point. Asterisks denote statistically significant values relative to the 0-time point (N = 3, Student’s *t*-test, *p* < 0.05).

Previously reported functional codon-optimized (L1.3) inducible human L1 (L1 EN+RT+) [57] was modified to contain inactivating mutations in either the EN (D205A), RT (D702A), or both domains [47]. These constructs are referred to as L1 EN−RT+, L1 EN+RT−, and L1 EN−RT−, respectively. Tet HEK 293T cells harboring these respective constructs were generated and induced by the addition of doxycycline (Dox, 1 µL/mL) as previously reported [57]. The accumulation of L1 ORF1 and ORF2 proteins, as well as the γH2AX signal, was monitored by Western blot analysis of total protein lysates collected from these cells at 3, 24, and 48 h post-induction (Figure 2). Western blot analysis performed using anti-ORF1p-specific antibodies, as previously described, demonstrated that all four constructs produce similar levels of L1 ORF1 protein with low levels of the protein being detected as early as 3 h post-induction (Figure 2A, ORF1 panel). Similar to the ORF1p, low levels of the ORF2 protein were also detected at 3 h post-induction using Western blot analysis with Flag-specific antibody (Figure 2A, ORF2 panel). Using this approach, several variants of the ORF2p were detected. This observation is in agreement with previous reports [32]. The detected variants are consistent with the ORF2 proteins that have lost their N-terminal sequences based on the size of detected bands and the presence of the Flag-tag on the C-terminus of the protein. A different trend in the ORF1p and ORF2p accumulation is observed between constructs expressing ORF2p with functional versus nonfunctional RT. Expression of ORF2p containing functional RT peaked at 24 h post-induction, while expression of ORF2p containing nonfunctional RT was comparable at 24 and 48 h post-induction (Figure 2).

It was previously reported that the expression of functional L1 elements in mammalian cells results in H2AX phosphorylation [65], which is indicative of the cellular response to DNA damage, such as DNA double-strand breaks and other types of lesions. Western blot analysis using anti-139 phospho-serine H2AX antibodies demonstrated accumulation of the γH2AX signal in cells induced to express L1 EN+RT+ or L1 EN-RT+ full-length elements (Figure 2). No significant increase in the γH2AX signal was observed when L1 EN+RT- or L1 EN-RT- full-length elements were expressed (Figure 2B).

### 3.2. RT-Dependent Accumulation of γH2AX Signal upon Induction of ORF2p Expression

**Figure 3:**
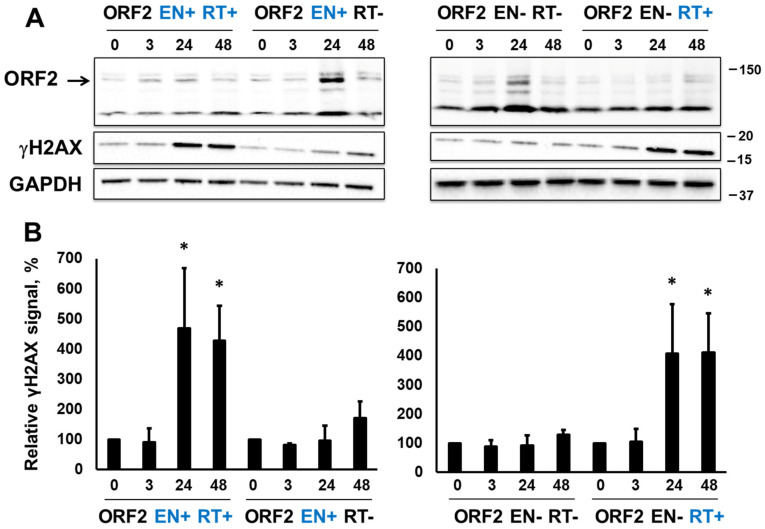
Expression of retrotransposition-incompetent ORF2 proteins triggers DNA damage response. (**A**) Western blot analysis of the ORF2 protein expression and γH2AX signal in HEK 293 cells at 3, 24, and 48 h post-induction of ORF2p expression with doxycycline. EN+ (blue) and EN− correspond to either functional or mutant endonuclease domains. RT+ (blue) and RT− indicate either functional or mutant reverse transcriptase domain. ORF2-specific antibodies were used to detect the ORF2 protein. A band corresponding to the ORF2p is indicated by the arrow. (**B**) Quantitation of the γH2AX signal relative to the 0-time point. Asterisks denote statistically significant values relative to the 0 time point (N = 3, Student’s *t*-test, *p* < 0.05).

To determine whether this pattern of γH2AX signal accumulation is unique to the full-length L1 element or whether it is specific to the L1 ORF2 protein, Tet HEK 293T cells harboring inducible constructs designed to express full-length functional human ORF2 protein (ORF2 EN+RT+) or ORF2 proteins containing inactivating mutations in their EN (D2015A), RT (D702A), or both the EN and the RT domains were generated. These constructs are referred to as ORF2 EN−RT+, ORF2 EN+RT−, and ORF2 EN−RT−, respectively. Tet HEK 293T cells were induced to express the respective ORF2 proteins via the addition of doxycycline (1 µL/mL) as previously described [57]. The accumulation of these ORF2 proteins, as well as the γH2AX signal, was analyzed using the total protein lysates collected from these cells at 3, 24, and 48 h post-induction. The expression of ORF2 proteins was detected using Western blot analysis with anti-ORF2p antibodies (Figure 3). DNA from cells harboring various ORF2 constructs was extracted and used for ORF2-specific PCR, followed by sequencing to confirm the wild-type or mutant status of EN and/or RT domains for each inducible cell line. Similar to the results observed with the full-length L1, expression of the EN+RT+ and EN−RT+ ORF2 proteins resulted in the significant accumulation of the γH2AX signal detected at 24 and 48 h after the addition of Dox (Figure 3A, ORF2 and γH2AX panels, and Figure 3B). No increase above the background γH2AX signal was detected over the 48 h time course in the HEK 293 cells harboring the ORF2 EN+RT− or EN−RT− constructs.

To confirm that the RT function remains intact when the EN domain is inactivated, a LEAP assay was performed as previously described (37). This approach demonstrated that both the EN+RT+ and the EN−RT+ ORF2 proteins were able to synthesize cDNA when an exogenous primer was included in the reaction (Figure S1) and support varying levels of Alu retrotransposition in HeLa cells (Figure S2).

**Figure 4:**
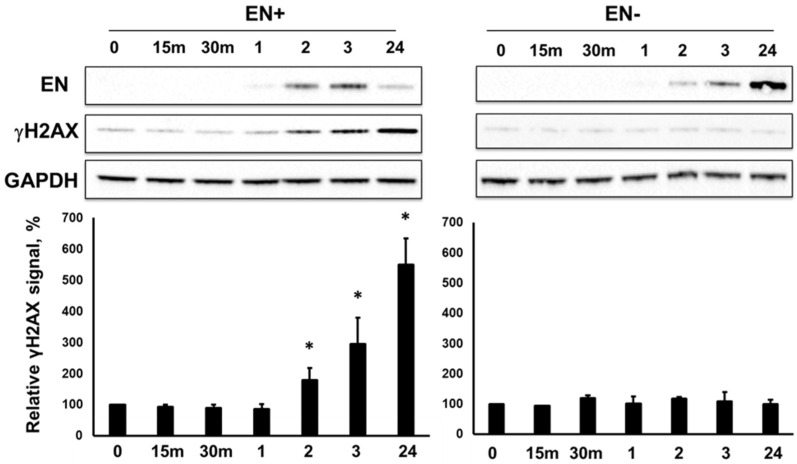
Expression of inducible EN protein triggers DNA damage response. Western blot analysis of the L1 endonuclease domain (EN) protein expression and γH2AX signal in HEK 293 cells at 15 munites (15 m), 30 minutes (30 m), 1, 2, 3, and 24 h post-induction of endonuclease expression with Doxycycline. EN+ and EN− indicate either a functional or mutant endonuclease. Anti-ORF2-specific antibodies were used to detect the EN+ and EN− proteins. Asterisks denote statistically significant values relative to the 0-time point (N = 3, Student’s *t*-test, *p* < 0.05).

**Figure 5:**
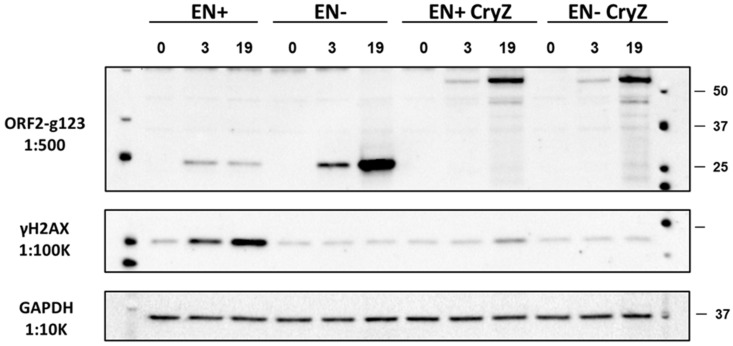
Inducible expression of C-terminally truncated ORF2p in HEK 293 cells. Constructs containing functional (EN+) or nonfunctional (EN−) endonuclease portions with or without the Cryptic (Cry) region of the human ORF2 sequence were generated for their use in the doxycycline-inducible system. Doxycycline treatment of HEK 293 cells containing these constructs resulted in the expression of corresponding proteins as detected by Western blot analysis with anti-ORF2p antibodies at 3 or 19 h after induction. Anti-phosphoH2AX-specific antibodies were used to detect the γH2AX signal. GAPDH detection was used as a loading control.

Although the induction of L1 EN+RT- or the ORF2 EN+RT- expression did not elicit significant DNA damage response (*p* = 0.06 or *p* = 0.07, respectively) as demonstrated by the lack of accumulation of the γH2AX signal (Figure 2 and Figure 3), the induction of the EN domain (aa 1–239) expression resulted in a significant increase in γH2AX signal as early as 2 h post-induction (Figure 4). As expected, the induction of the catalytically inactive EN domain did not elicit an increase in the γH2AX signal. We also confirmed that the Dox-driven expression of the C-terminally truncated ORF2p fragments, containing the endonuclease domain, Cryptic region, and Z-domain resulted in accumulation of the γH2AX signal only when the fragment, containing the functional endonuclease, was expressed (Figure 5**,** EN+CryZ). Consistent with our previous observations [32], the γH2AX signal in response to the expression of the EN+CryZ protein was reduced and delayed compared to the signal observed when the EN+ protein was expressed. No increase in the γH2AX signal was observed when the ORF2p fragment containing the nonfunctional EN (EN-CryZ) was expressed (Figure 5, EN-CryZ). The detection of the γH2AX signal as early as 2 h post-induction demonstrates the advantage of the inducible expression over transient transfections where the nonspecific DNA damage response masks cellular response to the L1-induced DNA damage during this time frame. The findings at 19 and 24 h are consistent with our previous results obtained using transient transfection of expression plasmids containing above mentioned fragments of the human ORF2 sequence [32].

### 3.3. L1 Expression Causes Large Genomic Deletions That Do Not Contain Any L1 Sequences

**Figure 6:**
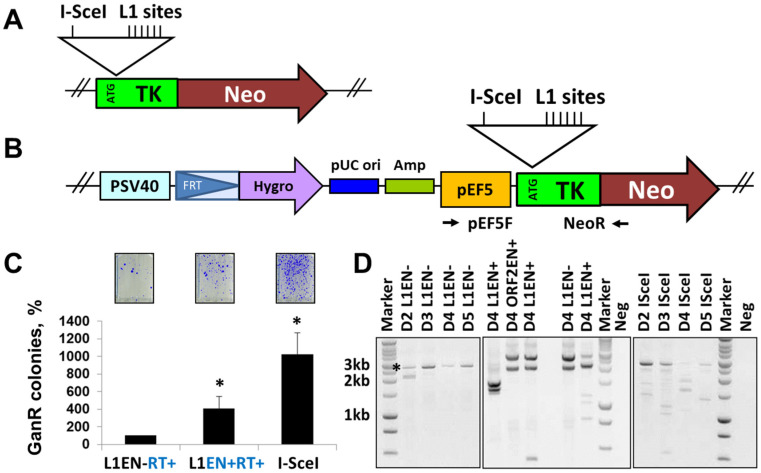
TK/Neo reporter system detects mutations resulting from the misrepair of L1-induced DNA breaks. (**A**) A codon-optimized sequence of thymidine kinase (TK) and neomycin resistance (Neo) was used to synthesize a fusion open reading frame containing in-frame I-SceI and L1 endonuclease (L1) site sequences. (**B**) Targeted TK/Neo cassette integration into HEK293 cells containing FRT site results in TK/Neo expression. (**C**) Outcome of ganciclovir selection following transfection with L1 EN-RT+ (labeled L1EN- in (**D**)), L1 EN+RT+ (labeled L1EN+ in (**D**)), or I-SceI expression plasmids. GanR are ganciclovir resistant colonies. Asterisks indicate statistical significance relative to the L1EN- (N = 3, Student’s *t*-test, *p* < 0.05). (**D**) Site-specific PCR analysis using primers (black arrows) complementary to the TK/Neo reporter cassette. D2–D5 are days post-transfection. Results of two independent experiments are shown in the middle panel. An asterisk indicates a 2.7 kb expected PCR product. Sequencing confirmed that larger PCR bands in the middle panel are nonspecific. Neg is negative no template control. Marker is a DNA ladder.

In addition to canonical L1 inserts that do not create any major structural rearrangements at their integration sites, de novo L1 integration sporadically causes large genomic deletions in cultured cells and in vivo [64,67]. These events are attributed to L1 because they contain L1 sequences and hallmarks of de novo L1 integration. To investigate the possibility that L1 could cause other types of genomic changes, including deletions or insertions that do not contain any signatures of L1 retrotransposition, we developed a reporter cassette designed to capture products of misrepaired L1-induced DSBs (Figure 6A). A series of L1 EN sites (5′-TTTTAA-3′) were included downstream of the translation initiation codon (ATG) of the thymidine kinase/neomycin (TK/Neo) fusion gene (Figure 6A and Figure S3). An I-SceI site is included as a positive control. This reporter cassette was integrated as a single copy into an FRT site that was previously inserted in the genome of HEK 293 FRT cells (Figure 6A,B, and Figure S3). Neomycin selection during propagation of clones with stably integrated TK/Neo cassette was used to prevent the accumulation of frameshift background mutations in this reporter sequence. Ganciclovir administration after transfection with plasmids expressing L1 (functional or EN mutant) or I-SceI allowed the survival of cells that have lost TK function due to the misrepair of DNA breaks (Figure S3). Formation of ganciclovir resistant (GanR) colonies resulting from the I-SceI induced DSBs was used as a positive control, L1 containing an inactivating mutation in the EN domain was used to control for the L1 endonuclease contribution to the observed signal (Figure 6C). This approach determined that the expression of functional L1 increases the frequency of GanR colonies compared to the expression of L1 containing an EN-inactivating mutation (Figure 6C).

To detect outcomes of misrepaired events within the TK/Neo cassette, we performed site-specific PCR using primers flanking the I-SceI, L1 site sequences in the TK/Neo cassette and DNA extracted from pooled GanR colonies (Supplemental Figure S4B). This approach identified 25 unique repair products out of 40 analyzed sequences recovered from the I-SceI-induced GanR colonies. These were deletions ranging from 1 to 8 bp or insertions ranging from 120 to 386 bp. This result is consistent with previous findings of I-SceI-induced small deletions and insertions [83]. All sequences recovered from the pooled L1EN- or L1 EN+ induced GanR colonies (N = 20 each) were either of the wild type or contained point mutations representing background. Site-specific PCR analysis of DNA extracted from individual L1 EN+-generated GanR colonies produces no PCR bands in most colonies screened by this approach (examples are shown in Supplemental Figure S4C). This result is consistent with the potential presence of deletions encompassing primer recognition sequences. The same result is observed when an ORF2p expression plasmid instead of the L1 expression plasmid was used for transient transfections (Supplemental Figure S5). Site-specific PCR with primers complementary to the reporter cassette sequences 5′ or 3′ distal to the L1 endonuclease sites identifies 1/6 colonies containing 5′ end sequences of the TK/Neo reporter and two of the same six colonies containing 3′ TK/Neo sequences (Supplemental Figure S5C). These findings support that exposure to wild-type L1 or ORF2p expression plasmids results in genomic deletions.

To capture some of these events, we performed site-specific PCR at 2–5 days post-transfection using primers that amplify 2.7 kb of the TK/Neo cassette. This site-specific PCR of genomic DNA extracted from cells transfected with indicated plasmids detects the expected 2.7 kb PCR product corresponding to the intact reporter cassette present in cells that have not yet died from the ganciclovir exposure, containing background mutations or containing small deletions/insertions that do not cause detectable change in the size of the PCR product (Figure 6D). This band is detected in cells transfected with I-SceI, functional, and EN-mutant L1 or ORF2p expression plasmids. The larger PCR band observed in the middle panel of Figure 6D is nonspecific. Sequencing analysis of 2.7 kb PCR products identified small deletions and insertions only in the PCR products generated using DNA extracted from cells transfected with the I-SceI expression plasmid.

No such events are detected in the PCR products generated using DNA extracted from cells exposed to L1 EN-, L1EN+, or ORF2EN+, i.e., these PCR products contained wt TK/Neo sequences or had random background point mutations inactivating TK function. This observation is consistent with our findings reflected in Supplemental Figure S4.

**Figure 7:**
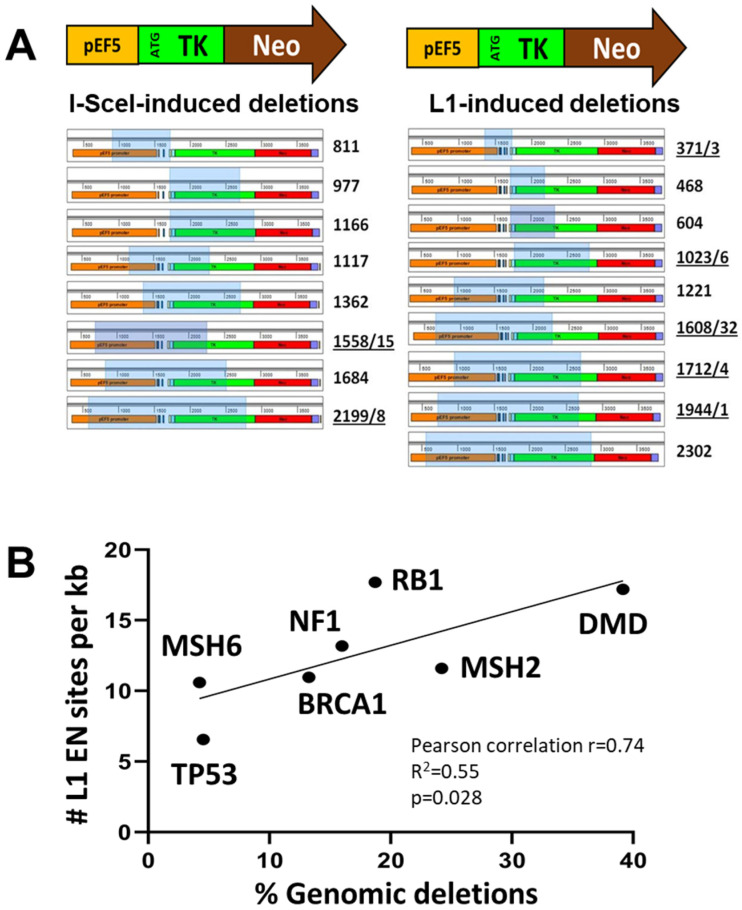
L1 expression causes large genomic deletions. (**A**) A schematic of the TK/Neo reporter cassette and deletions recovered from I-SceI (left) or L1-induced (right) cells. Numbers on the left of each panel indicate the size of deletion/number of non-template base pairs (underlined when occur together). Light blue boxes indicate deleted TK/Neo sequences. (**B**) Percent of genomic deletions correlates with the relative number of canonical L1 endonuclease sites. The number of canonical L1 endonuclease (EN) sites was identified in seven human genes and normalized to the gene lengths. The number of disease-causing deletions determined for the same genes correlates with the relative number of L1 EN sites in these genes (Pearson correlation r = 0.74). Kb is kilobase.

In addition to the 2.7 kb PCR product, this approach also detected smaller PCR products that are consistent with internal deletions in the TK/Neo cassette (Figure 6D). These products are observed in the cells transfected with plasmids expressing wild-type L1, ORF2p, or I-SceI. Sequencing analysis of smaller PCR products recovered from cells transfected with the plasmids expressing functional L1 identified deletions ranging from 300 to 2400 bp (Figure 6 and Figure 7A). 56% of these deletions (5/9) contained small non-template insertions (1–32 bp), which are also observed in 25% (2/8) of deletions recovered from the cells exposed to I-SceI. None of the deletions recovered from cells transfected with the wild-type L1 contain L1 sequences or A tail that could be consistent with L1 integration.

These findings support that L1 expression could be an underlying cause of at least some large genomic deletions that are currently not attributed to L1 because they do not contain any signatures of L1 involvement. To investigate a potential relationship between L1 EN sites and disease-causing deletions, we determined the frequency of canonical L1 EN sites in seven disease-relevant human genes: NF1, TP53, BRCA1, RB1, MSH6, MSH2, and DMD. The density of L1 EN sites per kb of gene sequence in these seven human genes strongly correlates with the relative frequency of large deletions cataloged for these genes in the human gene mutation database (Figure 7B, Pearson correlation r = 0.74, R^2^ = 0.55, *p* = 0.028).

### 3.4. L1 Endonuclease Cuts Telomeric Sequences In Vitro

**Figure 8:**
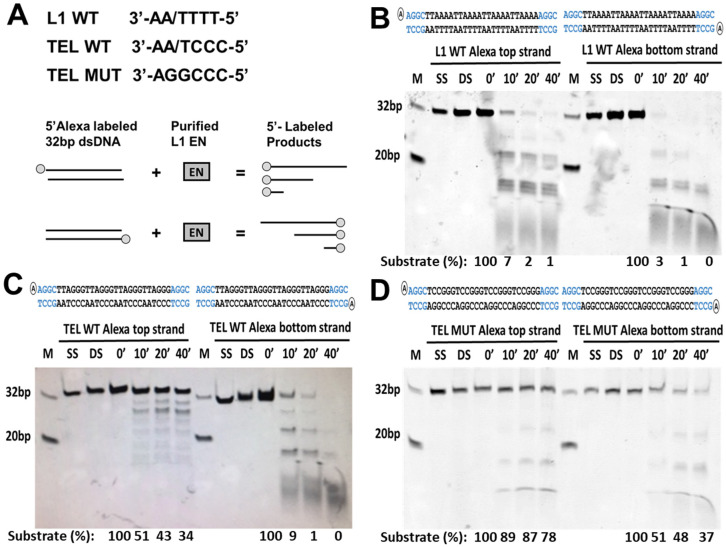
L1 endonuclease cuts telomeric repeat sequence in vitro. (**A**) Experimental design shows bottom strand sequences that are used to generate 5′ Alexa labeled 32 bp long double-stranded DNA. L1 WT is the L1 endonuclease consensus site, TEL WT is a wild wild-type telomeric repeat sequence, and TEL MUT is a mutant telomeric sequence. Overall, 5′ top or bottom strand labeled dsDNA was used as a substrate for a purified wild-type L1 endonuclease domain (EN) in an in vitro cleavage assay. Blue indicates L1 or TEL unrelated extra sequence included in design. Products were assessed after 10, 20, or 40 min of incubation. Resulting products are shown for L1 WT (**B**), TEL WT (**C**), and TEL MUT (**D**) substrates. Primer sequences are shown at the top of each panel. M is a 32 and 20 bp marker, SS single strand DNA, DS double-strand DNA, 0′, 10′, 20′, and 40′ are incubation times in minutes. Numbers at the bottom (Substrate %) represent the quantitation of the remaining substrate relative to the initiation time point (0′).

In addition to L1 endonuclease cleavage sites scattered throughout genomic DNA, telomeric repeat sequence (5′-TTAGGG-3′) is one of the noncanonical L1 endonuclease cleavage sites based on the reported L1 endonuclease consensus sequence [43,84]. To test whether L1 EN can cut this sequence, we used our previously reported in vitro endonuclease cleavage assay [68]. Functional L1 endonuclease domain purified from *E. coli* was used to test its ability to cut double-strand (ds) DNA containing canonical L1 endonuclease cleavage sites (5′-TTTT/AA-3′), wild-type (5′-TTAGGG-3′) or mutant (5′-TCCGGG-3′) telomeric repeats. The experiment was carried out with dsDNA containing 5′-end Alexa labeling on either top or bottom strands (Figure 8A). This approach shows that the L1 endonuclease efficiently cuts dsDNA containing canonical L1 EN sites and wild-type telomeric repeats (Figure 8B,C).

A time course analysis shows that within 10 min of the substrate incubation with L1 endonuclease over 90% of the dsDNA containing canonical L1 sites is cut at various sites (Figure 8B). Efficient cleavage occurs on both the top and bottom strands of the substrate (Figure 8B). Under the same experimental conditions, the TEL WT substrate is cleaved more efficiently on the bottom strand than the top strand, with 90% and 50% of the respective substrates being cut within 10 min of incubation with the L1 EN domain (Figure 8C, the chi-square statistic is 50.106. The *p*-value is <0.00001. The result is significant at *p* < 0.05). Chi-square analysis determined that the purified L1 endonuclease cuts the bottom strand of the TEL WT substrate as efficiently as it cuts the bottom strand of the L1 WT substrate (the chi-square statistic is 2.834. The *p*-value is 0.24244. The result is not significant at *p* < 0.05). However, the L1 WT top strand sequence is a significantly better L1 EN substrate than the top strand sequence of the TEL WT dsDNA (The chi-square statistic is 69.0724. The *p*-value is <0.00001. The result is significant at *p* < 0.05). While some cleavage of the TEL MUT substrate is observed after 10 min of incubation with the L1 EN, most of this DNA remains intact after 40 min of exposure to the enzyme compared to the L1 WT and TEL WT substrates (Figure 8, 78% top strand label versus 1 and 34%, respectively). Chi-square analysis confirmed that the TEL WT sequence is a significantly better substrate for L1 EN cleavage than the TEL MUT sequence (top strand label, the chi-square statistic is 15.9621. The *p*-value is 0.001154. The result is significant at *p* < 0.05, and bottom strand label, the chi-square statistic is 53.2662. The *p*-value is <0.00001. The result is significant at *p* < 0.05). Despite being an overall poor substrate, the bottom strand sequence of the TEL MUT dsDNA is a better substrate for the L1 EN cleavage than the top strand of the TEL MUT substrate (Figure 8D, the chi-square statistic is 13.1233. The *p*-value is 0.004377. The result is significant at *p* < 0.05). This result is consistent with the findings observed for the TEL WT substrate (Figure 8C).

## 4. Discussion

It has been demonstrated that L1 expression in the germ line, during embryogenesis, and in adult somatic tissues contributes to genomic instability via the generation of de novo L1 inserts [64,85,86]. L1 mRNA and protein expression, as well as L1 retrotransposition, are significantly increased in most human tumors and cancer cell lines [16,20]. Although both functional and nonfunctional L1 loci are expressed in human, mouse, and rat cells and organs [9,10,27,87], the potential impact from the retrotransposition-incompetent L1 loci has only recently been recognized [30,31,32,33]. Similarly, recent findings support that L1 retrotransposition is only one of the potentially many ways expression of functional L1 loci may affect host genome stability or cellular function [63,65,88,89].

We identified that the induced expression of retrotransposition-incompetent L1 or ORF2p containing a nonfunctional EN domain elicits the accumulation of γH2AX signal in cultured mammalian cells. This phenomenon is associated with the functional RT of the L1 ORF2p (Figure 2 and Figure 3) because γH2AX signal accumulation is observed when the expression of EN+RT+ or EN-RT+, but not EN+RT- ORF2p, is induced (Figure 2 and Figure 3). These findings suggest that the EN function may be limited in the full-length ORF2p in order to be used in a coordinated manner during other steps of the retrotransposition cycle. This possibility is supported by the robust accumulation of the γH2AX signal in cells expressing functional L1 endonuclease domain (Figure 4 and Figure 5). This response to EN expression is notably dampened when an ORF2p fragment containing the EN, Cry, and Z sequences is expressed, even though the ENCryZ protein accumulates to higher steady-state levels than the EN protein by 19 h post-induction (Figure 5). This observation is consistent with our previously published data [32,69] that the Cryptic sequence within the human ORF2p may suppress L1 endonuclease function via a yet unknown mechanism. The lack of detectable increase in the γH2AX signal in cells expressing L1 or ORF2p EN+RT- (Figure 2 and Figure 3) could be explained by either rapid repair of the EN-induced nicks in the absence of the functional RT or by the aforementioned suppression of EN activity in the context of the full-length ORF2 protein lacking a functional RT as previously suggested [69]. It is worth noting that the two scenarios are not mutually exclusive.

We speculate that the RT-induced DNA damage response may result from the ORF2p’s ability to utilize the EN-induced or existing DNA breaks for cDNA synthesis. This possibility is supported by the published observations that EN-deficient L1 elements retrotranspose in NHEJ DNA-repair-deficient cells [31] as well as our observations that EN-RT+ ORF2p retains its ability to make cDNA, using ORF2 mRNA, and supports residual Alu retrotransposition in HeLa cells (Supplemental Figure S2 and [31,40]). This finding is also consistent with the ability of truncated ORF2 proteins lacking the EN domain to drive low levels of Alu retrotransposition in HeLa cells [40]. The EN-RT+ ORF2p association with existing single or double-strand DNA breaks and resulting cDNA synthesis could lead to DNA damage response to resolve these stalled retrotransposition intermediates. Alternatively, an association of the EN-mutant ORF2p with existing nicks or DSBs may impede their repair, leading to detectable DNA damage response. This DDR can help complete or interfere with the initiated integration [90,91,92]. The discovery of the RT-mediated DDR is also consistent with the previous observation that only when the RT function is mutated, the ORF2 mediated toxicity is completely eliminated [70]. The finding that RT alone is sufficient to trigger DDR is yet another way by which retrotransposition-incompetent L1 loci may perturb cellular function. The consequences of this outcome could be chronic DDR that may contribute to the inflammatory response attributed to the L1 RT-mediated cDNA synthesis (Figure 1) because chronic DNA damage can lead to an inflammatory response [93].

L1 elements containing mutant EN can retrotranspose in telomeres of cells deficient in DNA-PKcs function [30]. It is established that L1 EN cuts AT-rich sequences in vitro and in vivo [43]; however, whether L1 EN can cut telomeric repeats is not known. It is also not known whether L1 EN-induced DNA breaks result in mutations, and if so, the spectrum of these mutations is likewise unknown. We provide evidence that L1 expression leads to a measurable increase in mutagenic events (Figure 4, Figures S4 and S5) and that misrepair of L1-induced DNA breaks in HEK 293 cells produces large genomic deletions (Figure 6 and Figure 7). In contrast to the deletions associated with L1 integration, these deletions do not contain any traceable L1 sequences that would indicate L1 involvement. We cannot rule out the possibility that these events result from aborted L1 retrotransposition intermediates, repair of which eliminated all hallmarks of L1 involvement. However, it is also possible that the repair of L1-induced DNA breaks occurred prior to or without initiation of cDNA synthesis. Our findings support that the generation of these deletions is L1-EN-dependent (Figure 6C,D). In contrast to the I-SceI-induced DSBs, which frequently result in small deletions/insertions, small in/dels have not been detected at the L1 sites in our reporter cassette. There are several plausible explanations for this difference in the I-SceI and L1 mutational spectrum. One possibility is the difference in the nature of the breaks induced by these two enzymes. I-SceI produces a 5′ overhang DSBs. The structure of the L1-induced breaks in vivo is not known. L1-generated DNA nicks may become DSBs during cell division. Alternatively, L1-induced DNA breaks can be bulky DNA lesions containing newly synthesized cDNA. Another possibility for the difference in the I-SceI and L1 mutation spectrum is the number of proximal breaks. Only one copy of the I-SceI recognition sequence is present in our reporter system and the genome. In contrast, multiple canonical L1 EN recognition sites are introduced in the TK/Neo reporter sequence. Additionally, multiple canonical and noncanonical L1 EN recognition sites are present in the sequence surrounding our reporter cassette. Thus, it is possible that deletions detected after exposure to L1 result from the misrepair of multiple L1-induced DNA breaks in the region rather than from a single L1-induced DNA break. While further studies to understand the mechanism underlying L1-induced “genomic scars” are needed, our findings establish the possibility that chronic L1 expression in the germline, as well as normal and cancer cells, can contribute to genomic deletions that have no traceable marks of L1 involvement.

These findings call for the reassessment of an already significant L1 contribution to evolution [94,95] and human disease [64,66]. For example, up to 35% of somatically acquired mutations in the genome of an average human cancer sample are represented by large or focal deletions [96], some of which are responsible for the origin or progression of the disease [97]. DSB repair induced genomic deletions underly chromoplexy, which is recognized to drive prostate cancer progression to its aggressive form [98]. None of these deletions are currently attributed to L1, but based on our findings some of them could be generated by L1 because L1 expression in cancer correlates with copy number alterations [99]. A higher genomic content of full-length L1 loci is reported in African American (AA) populations compared to other populations [100]. Coincidently, AAs have a higher rate and/or more aggressive cancers compared to the Caucasian population [101]. L1-induced deletions could also occur in the germline, where high levels of L1 expression are observed. Cytogenetically detectable germline deletions occur once in every 7000 live births [102]. They could also contribute to a significant number of idiopathic infertility cases and spontaneous abortions. Supporting the possibility of L1 contribution to disease-causing deletions, we observe a strong correlation between the density of L1 sites within seven human genes and the frequency of the disease-causing deletions in these genes (Figure 7B). In addition to genomic deletions, we demonstrate that L1 endonuclease can cut telomeric sequences in vitro almost as efficiently as it cleaves canonical L1 sites (Figure 8). While it remains uncertain whether L1 can cut telomeres in vivo, this observation provides strong support for testing the hypothesis that L1-induced breaks may be contributing to telomere shortening during aging. If it were true, L1-induced DNA breaks at telomeres might be one of the reasons behind the short telomeres observed in telomerase-expressing cancer cells where L1 expression is typically elevated [103].

## 5. Conclusions

In summary, we provide evidence that in addition to retrotransposition, the expression of functional and nonfunctional L1 loci may adversely affect host genome stability or cellular function by triggering RT-mediated DNA damage response, causing large genomic deletions or telomere dysfunction. Our findings expand the spectrum of mutations associated with L1 expression by identifying deletions that do not contain any hallmarks of L1 involvement. These results support the need to understand the extent of L1 contribution to genomic deletions that drive genome evolution, accumulate with age, and contribute to human diseases, including cancer.

## Data Availability

Data are contained within the article and Supplementary Materials.

## Supplementary Materials

The following supporting information can be downloaded at: https://www.mdpi.com/article/10.3390/genes15020143/s1.
