# Supplementary material for: Large Deletions, Cleavage of the Telomeric Repeat Sequence, and Reverse Transcriptase-Mediated DNA Damage Response Associated with Long Interspersed Element-1 ORF2p Enzymatic Activities
Source: Genes (Basel). 2024 Jan 23;15(2):143. doi: 10.3390/genes15020143 (PMC10887698; doi:10.3390/genes15020143)
Supplement: Supplementary file 1 [file genes-15-00143-s001.zip › genes-2814616-supplementary.pdf]

Supplemental Materials: The following supportive information can be found Figures S1–7.

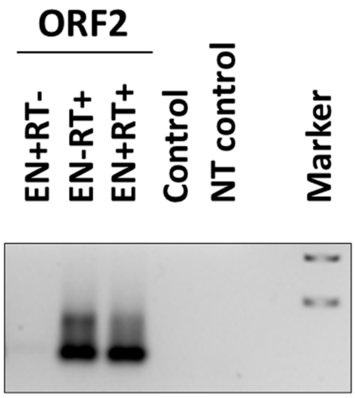

Figure S1. ORF2 proteins containing inactive endonuclease retain the ability to synthesize cDNA in HEK 293 cells. ORF2 expression plasmids designed to express either a wild type (EN+RT+) or mutant (EN+RT- or EN-RT+) proteins were transiently transfected into HEK 293 cells. The polyribosomal fraction was purified 24 hours after transfection and used for assessing their ability to perform RT-dependent cDNA synthesis as previously described. The resulting DNA products were fractionated on an agarose gel. Control are cellular lysates from cells transfected with an empty plasmid. NT control is a no template control during PCR amplification of the cDNA products. Marker indicates a DNA ladder.

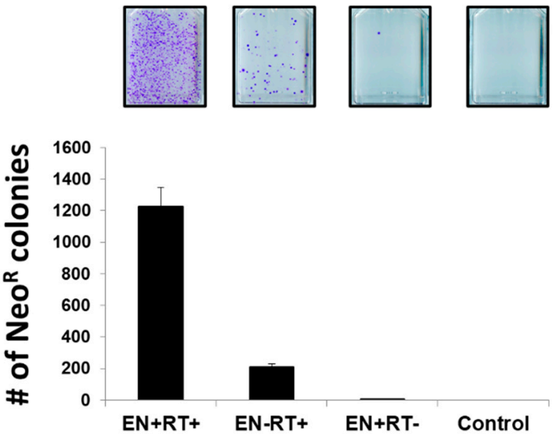

Figure S2. EN-RT+ ORF2p supports low levels of Alu retrotransposition in HeLa cells. Constructs containing EN+RT+, EN+RT-, or EN-RT+ ORF2 sequences and AluNeo expression construct were transiently cotransfected into HeLa cells. G418 selection for 14 days resulted in Neo-resistant colonies formed by cells harboring *de novo* Alu integration events. Alu retrotransposition supported by EN-RT+ was significantly lower than the control (EN+RT+) (N=3, student's t-test, $p<0.05$).

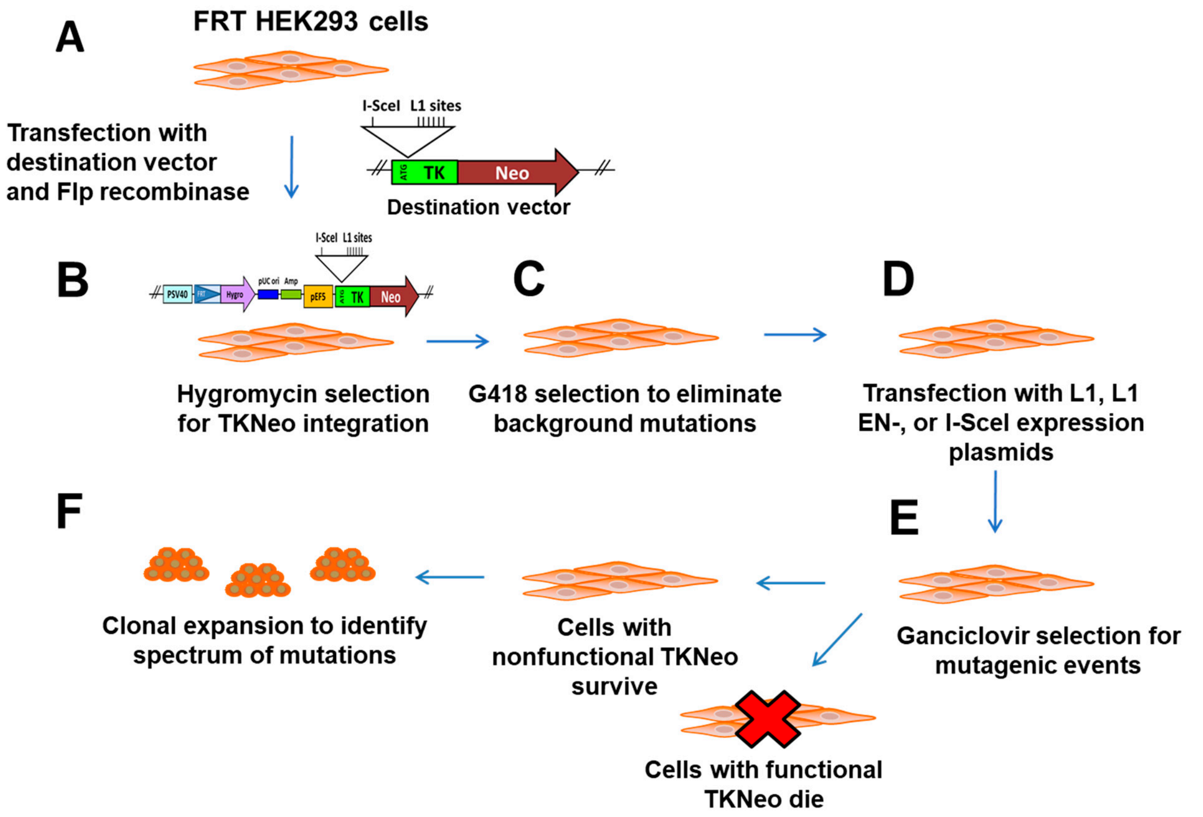

Figure S3. Schematic of the experimental approach to generate HEK 293 cells harboring a single copy of the TK/Neo reporter cassette. A. Existing FRT HEK 293 cells harboring a single copy of the FRT cite are transiently co-transfected with a destination plasmid containing TK/Neo sequence and Flp recombinase expression plasmid. **B.** Flp-mediated recombination results in hygromycin resistance (HygR). **C.** HygR cells are next selected with G418 to eliminate cells that may have accumulated random mutations in the reporter cassette. **D.** Transient transfection of NeoR cells with plasmids of interest followed by Gan selection to capture cells containing mutagenic TK events (**E**). **F.** Resulting GanR colonies can be used for analysis of mutagenic events.

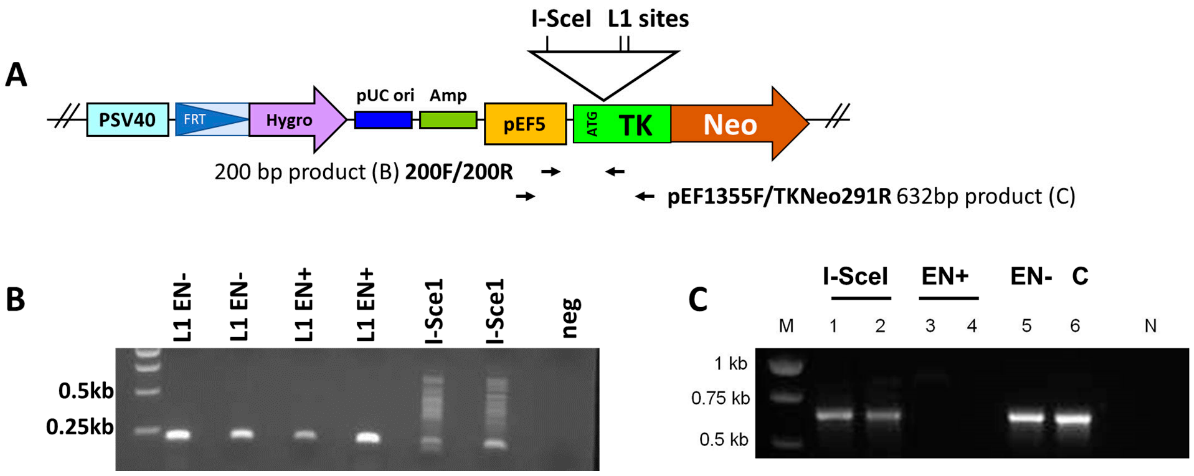

Figure S4. Analysis of mutations induced by transient L1 expression. A. Schematic of the integrated TK/Neo reporter cassette. Black horizontal arrows indicate approximate position of PCR primers and product size. **B.** Site-specific PCR analysis of DNA extracted from pulled GanR colonies resulting from exposure to L1 EN- (mutant EN), L1 EN+ (functional EN), or I-SceI (N=2). Neg is negative control is original FRT HEK 293 cells that do not contain the TK/Neo cassette. **C.** Site-specific PCR analysis of individual GanR colonies resulting from exposure of FRT HEK 293 or NIH-3T3 cells to I-SceI (1 and 2), L1 EN+ (3 and 4), or L1 EN- (6). C is a positive control for the TK/Neo cassette detection in the HEK 293 cells DNA before Gan selection. N is no template control.

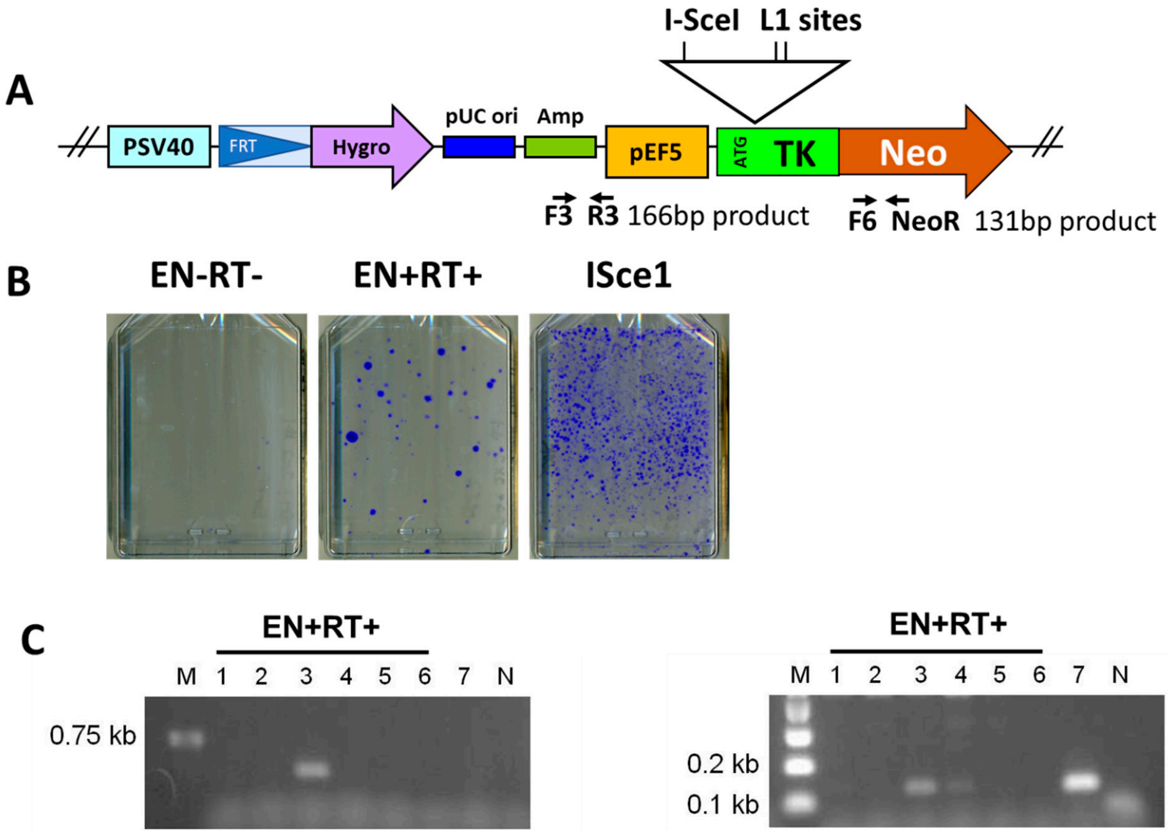

Figure S5. ORF2p expression causes large deletions. A. Schematic of the TK/Neo cassette integrated in the FRT site of HEK 293 cells. Black horizontal arrows indicate approximate positions of PCR primers. **B.** GanR colonies resulting from TK/Neo HEK 293 transfection with plasmids expressing EN-RT-, EN+RT+ ORF2p, or I-SceI. **C.** Site-specific PCR analysis of DNA extracted from 6 GanR colonies (Lanes 1-6) using primer pairs complementary to sequences 5' (left) and 3' (right) of the L1 sites. N is no template control. Lane 7 genomic DNA from FRT HEK 293 cells without TK/Neo. The F3/R3 and F6/NeoR primer sets are not expected to generate PCR products when DNA extracted from the FRT HEK 293 cells is used as a template. The band in Lane 7 (right panel) is nonspecific.

File S1. Annotated sequence of the TK/Neo reporter cassette.

pEF5 Promoter (pEF5F primer, pEF1355F primer, 200F primer)

CGTGAGGCTCCGGTGCCCGTCAGTGGGCAGAGCGCACATCGCCCACAGTCCCCGAGAAGTTGGGGGGAGGGGT
CGGCAATTGAACCGGTGCCTAGAGAAGGTGGCGCGGGGTAAACTGGGAAAGTGATGTCGTGTACTGGCTCCGC
CTTTTTCCCGAGGGTGGGGGAGAACCGTATATAAGTGCAGTAGTCGCCGTGAACGTTCTTTTTCGCAACGGGTTT
GCCGCCAGAACACAGGTAAGTGCCGTGTGTGGTTCCCGCGGGCCTGGCCTCTTTACGGGTTATGGCCCTTGCGT
GCCTTGAATTACTTCCACCTGGCTGCAGTACGTGATTCTTGATCCCGAGCTTCGGGTTGGAAGTGGGTGGGAGA
GTTCGAGGCCTTGCGCTTAAGGAGCCCCTTCGCCTCGTGCTTGAGTTGAGGCCTGGCCTGGGCGCTGGGGCCGC
CGCGTGCGAATCTGGTGGCACCTTCGCGCCTGTCTCGCTGCTTTCGATAAGTCTCTAGCCATTTAAAATTTTTGAT
GACCTGCTGCGACGCTTTTTTTCTGGCAAGATAGTCTTGTAAATGCGGGCCAAGATCTGCACACTGGTATTTCGG
TTTTTGGGGCCGCGGGCGGCGACGGGGCCCGTGCGTCCCAGCGCACATGTTCGGCGAGGCGGGGCCTGCGAGC
GCGGCCACCGAGAATCGGACGGGGGTAGTCTCAAGCTGGCCGGCCTGCTCTGGTGCCTGGCCTCGCGCCGCCGT
GTATCGCCCCGCCCTGGGCGGCAAGGCTGGCCCGGTCGGCACCAGTTGCGTGAGCGGAAAGATGGCCGCTTCCC
GGCCCTGCTGCAGGGAGCTCAAAATGGAGGACGCGGCGCTCGGGAGAGCGGGCGGGTGAGTCACCCACACAA
AGGAAAAGGGCCTTTCCGTCCTCAGCCGTCGCTTCATGTGACTCCACGGAGTACCGGGCGCCGTCCAGGCACCTC
GATTAGTTCTCGAGCTTTTGGAGTACGTCGTCTTTAGGTTGGGGGGAGGGGTTTTATGCGATGGAGTTTCCCCAC
ACTGAGTGGGTGGAGACTGAAGTTAGGCCAGCTTGGCACTTGATGTAATTCTCCTTGGAATTTGCCCTTTTTGAG
TTTGGATCTTGGTTCATTCTCAAGCCTCAGACAGTGGTTCAAAGTTTTTTTCTTCCATTTCAGGTGTCGTGA
GGAATTAGCTTGGTACTAATACGACTCACTATAGGGAGACCCAAGCTGGCTAGGTAAGCTTGGTACCGAGCTCGG
ATCCACTAGTCCAGTGTGGTGGAATTCTGCAGATATCAACAAGTTTGTACAAAAAAGCAGGcaccggaAGATCTtcgc
cgccacc

HSV TK (I-SceI site, L1 sites, 200R primer, TK/Neo295R primer,)

ATGGCTGCTAGCctatattaccctgttaTCcctagcgtaactGCTAGCattttaagaatttttattttaaattttcttttaaaattttaattaat
TCGTACCCCTGCCATCAACATGCGTCTGCGTTCGACCAGGCTGCGCGTTCTCGCGGCCATAACAACCGACGTACGG
CGTTGCGCCCTCGCCGGCAACAAAAAGCCACGGAAGTCCGCCTGGAGCAGAAAATGCCCACGCTACTGCGGGTTT
ATATAGACGGTCCCCACGGGATGGGGAAAACCACCACCACGCAACTGCTGGTGGCCCTGGGTTCGCGCGACGAT
ATCGTCTACGTACCCGAGCCGATGACTTACTGGCGGGTGTTGGGGGCTTCCGAGACAATCGCGAACATCTACACC
ACACAACACCGCCTCGACCAGGGTGAGATATCGGCCGGGGACGCGGCGGTGGTAATGACAAGCGCCCAGATAAC
AATGGGCATGCCTTATGCCGTGACCGACGCCGTTCTGGCTCCTCATATCGGGGGGGAGGCTGGGAGCTCACATGC
CCCGCCCCCGGCCCTCACCCTCATCTTCGACCGCCATCCCATCGCCGCCCTCCTGTGCTACCCGGCCGCGCGATACC
TTATGGGCAGCATGACCCCCCAGGCCGTGCTGGCGTTCGTGGCCCTCATCCCGCCGACCTTGCCCGGCACAAACAT
CGTGTTGGGGGCCCTTCCGGAGGACAGACACATCGACCGCCTGGCCAAACGCCAGCGCCCCGGCGAGCGGCTTG
ACCTGGCTATGCTGGCCGCGATTCGCCGCGTTTATGGGCTGCTTGCCAATACGGTGCGGTATCTGCAGGGCGGCG
GGTCGTGGCGGGAGGATTGGGGACAGCTTTCGGGGGCGGCCGTGCCGCCCCAGGGTGCCGAGCCCCAGAGCAA
CGCGGGCCCACGACCCCATATCGGGGACACGTTATTTACCCTGTTTCGGGCCCCCGAGTTGCTGGCCCCCAACGGC
GACCTGTATAACGTGTTTGCCTGGGCTTTGGACGTCTTGGCCAAACGCCTCCGTCCCATGCATGTCTTTATCCTGGA
TTACGACCAATCGCCCGCCGGCTGCCGGGACGCCCTGCTGCAACTTACCTCCGGGATGGTCCAGACCCACGTCACC
ACCCCAGGCTCCATACCGACGATCTGCGACCTGGCGCGCACGTTTGCCCGGGAGATGGGGGAGGCTAAC

Neo (F6 primer, NeoR primer)

ATTGAACAAGATGGATTGCACGCAGGTTCTCCGGCCGCTTGGGTGGAGAGGCTATTCGGCTATGACTGGGCACA
ACAGACAATCGGCTGCTCTGATGCCGCCGTGTTCCGGCTGTCAGCGCAGGGGCGCCCGGTTCTTTTTGTCAAGAC
CGACCTGTCCGGTGCCCTGAATGAACTGCAGGACGAGGCAGCGCGGCTATCGTGGCTGGCCACGACGGGCGTTC
CTTGCGCAGCTGTGCTCGACGTTGTCACTGAAGCGGGAAGGGACTGGCTGCTATTGGGCGAAGTGCCGGGGCAG
GATCTCCTGTCATCTCACCTTGCTCCTGCCGAGAAAGTATCCATCATGGCTGATGCAATGCGGCGGCTGCATACGC
TTGATCCGGCTACCTGCCCATTCGACCACCAAGCGAAACATCGCATCGAGCGAGCACGTACTCGGATGGAAGCCG
GTCTTGTCGATCAGGATGATCTGGACGAAGAGCATCAGGGGCTCGCGCCAGCCGAACTGTTCGCCAGGCTCAAGG
CGCGCATGCCCGACGGCGAGGATCTCGTCGTGACCCATGGCGATGCCTGCTTGCCGAATATCATGGTGGAAAATG

GCCGCTTTTCTGGATTCATCGACTGTGGCCGGCTGGGTGTGGCGGACCGCTATCAGGACATAGCGTTGGCTACCC
GTGATATTGCTGAAGAGCTTGGCGGCGAATGGGCTGACCGCTTCCTCGTGCTTTACGGTATCGCCGCTCCCGATTC
GCAGCGCATCGCCTTCTATCGCCTTCTTGACGAGTTCTTCTGA
CGGAGTACTGTCCTCCGAGCGGAGTACTGTCCTCCGACTCGAGCGGAGTACTGTCCTCCGATCGGAGTACTGTCCT
CCGCGAATTCCGGAGTACTGTCCTCCatgcatACCCAGCTTTCTTGTACAAAGTGGTTGATATCCAGCACAGTGGCGG
CCGCTCGAGTCTAGAGGGCCCGCGGTTCGAAGGTAAGCCTATCCCTAACCCTCTCCTCGGTCTCGATTCTACGCGT
ACCGGTTAGTAATGAGTTTAAACCCGCTGATCAGCCTCGA

BGH polyadenylation signal

CTGTGCCTTCTAGTTGCCAGCCATCTGTTGTTTGCCCCTCCCCCGTGCCTTCCTTGACCCTGGAAGGTGCCACTCCCA
CTGTCCTTTCCTAATAAAATGAGGAAATTGCATCGCATTGTCTGAGTAGGTGTCATTCTATTCTGGGGGGTGGGGT
GGGGCAGGACAGCAAGGGGGAGGATTGGGAAGACAATAGCAGGCATGCTGGGGATGCGGTGGGCTCTATGG
